# Eligibility of Dapagliflozin and Empagliflozin in a Real-World Heart Failure Population

**DOI:** 10.1155/2021/1894155

**Published:** 2021-12-26

**Authors:** Erik Håkansson, Helena Norberg, Sara Själander, Krister Lindmark

**Affiliations:** ^1^Department of Public Health and Clinical Medicine, Umeå University, S-901 87 Umeå, Sweden; ^2^Department of Pharmacology and Clinical Neuroscience, Department of Public Health and Clinical Medicine, Umeå University, S-901 87 Umeå, Sweden

## Abstract

**Aims:**

This study is aimed at investigating the eligibility in a real-world heart failure population for the DAPA-HF (testing dapagliflozin) and EMPEROR-reduced (testing empagliflozin) trials, comparing the eligible real-world patients to trial participants and to characterize the noneligible patients.

**Methods:**

Medical records of all heart failure patients who had a diagnosis of heart failure from the Heart Centre or Department of Internal Medicine at Umeå University Hospital were reviewed.

**Results:**

2433 of the hospital's uptake population of 150 000 had a diagnosis of heart failure. 681 patients had left ventricle ejection fraction ≤ 40%, and of these 352 (52%) and 268 (39%) patients met eligibility criteria for DAPA-HF and EMPEROR-reduced, respectively. Comparing eligible patients in our population with the DAPA-HF- and EMPEROR-reduced trial populations, we found that eligible real-world patients were older (79.0 vs. 66.2 years and 80.3 vs. 67.2 years, respectively), had worse renal function (eGFR 54.4 vs. 66.0 ml/min/1.73m^2^ and 49.5 vs. 61.8 ml/min/1.73m^2^, respectively), higher prevalence of atrial fibrillation (56.0% vs. 36.1% and 53.0% vs. 35.6%, respectively), and lower prevalence of diabetes mellitus (21.0% vs. 41.8% and 26.1% vs. 49.8%, respectively). The main reasons for ineligibility were low NT-proBNP or low eGFR. Noneligible patients differed according to reason for ineligibility, where patients with low NT-proBNP were generally younger and healthier, and patients with low eGFR were older and had more comorbidities.

**Conclusions:**

39-52% of patients with heart failure and reduced ejection fraction in this real-world heart failure population were eligible for SGLT2-inhibitor treatment, corresponding to 11-14% of all heart failure patients. Compared to trial participants, eligible real-world patients were significantly older with worse renal function, more atrial fibrillation, and less diabetes mellitus. Trial entry criteria exclude comparatively young and healthy patients, as well as comparatively old patients with more comorbid conditions.

## 1. Introduction

Heart failure (HF) is a common condition with poor prognosis [1]. Guideline-based treatment for heart failure with reduced ejection fraction (HFrEF) includes pharmacological treatment with an angiotensin converting enzyme inhibitor (ACE-I) or angiotensin receptor blocker (ARB), sometimes together with a neprilysin inhibitor (ARNI), a beta blocker, and a mineralocorticoid receptor antagonist (MRA), as well as implantable cardiac devices such as cardiac resynchronization therapy (CRT) and implantable cardioverter-defibrillators (ICD) [2]. Despite all these treatment options, mortality and morbidity remain high. In addition to premature deaths and decreased quality of life, patients with HF represent a significant cost to healthcare systems [3]. New treatments are needed.

Drugs used in diabetes mellitus (DM) that block the sodium-glucose cotransporter 2 (SGLT2) have shown reduced HF-hospitalizations among patients treated for type 2 diabetes mellitus (T2DM) [4–6]. These findings spawned several clinical trials designed to study SGLT2-inhibitors in HF with or without DM. Two of these trials aimed at HFrEF have been published: DAPA-HF studying dapagliflozin [7] and EMPEROR-reduced studying empagliflozin [8]. Both trials demonstrated a reduction in HF hospitalizations and death from cardiovascular causes that was independent of DM status. However, randomized controlled trials (RCTs) commonly study a selected population which does not necessarily reflect the real-world population in which the studied intervention is meant to be used [9]. This can reduce their applicability in the clinic. Furthermore, DAPA-HF and EMPEROR-reduced had slightly different inclusion and exclusion criteria making them less immediately comparable. With this study, we aim to investigate how comparable the DAPA-HF and EMPEROR-reduced populations are with a real-world HF population. We will address the following questions:

- What proportion of a real-world HF population would have been eligible for dapagliflozin and/or empagliflozin, according to the main inclusion and exclusion criteria used in the DAPA-HF and EMPEROR-reduced trials?
- How comparable are the DAPA-HF and EMPEROR-reduced populations to eligible real-world patients?
- What characterizes patients with HFrEF who are not eligible for SGLT2-inhibitor treatment according to the main inclusion and exclusion criteria used in the DAPA-HF and EMPEROR-reduced trials?

## 2. Materials and Methods

### 2.1. Study Population

We retrospectively studied all patients aged 18 years or older who had had at least one contact with the Heart Centre or Department of Internal Medicine at Umeå University Hospital, Sweden, between January 2010 and December 2019 and received a diagnosis of HF (10^th^ revision of the *International Classification of Disease and Related Health Problems* codes I50.X, I42.0, I46.6, I42.7, I42.9, I11.0, I13.0, I13.2) and lived within the catchment area of the hospital. Any contact with a diagnosis attached was counted, i.e., outpatient visit, emergency visit, or inpatient stay. The hospital serves a mixed urban and rural population with roughly 150 000 inhabitants. The hospital is the only hospital within the catchment area and thus has the area's only cardiology clinic and internal medicine clinic.

### 2.2. Data Collection

We used a prespecified protocol to manually extract data from the hospital's electronic medical records system (NCS Cross). The protocol contained variables about medical history, electrocardiogram and echocardiogram parameters, lab results, common comorbidities, and prescribed medication. Record entries from the Heart Centre and Department of Internal Medicine were checked for applicable parameters. Patients were coded as having atrial fibrillation, hypertension, or diabetes if there was a matching ICD-10 code for respective condition. The heart failure etiology was coded as ischemic if the patient had a history of myocardial infarction, revascularization for ischemic heart disease, or coronary vessel stenosis of >50% on coronary angiography. Patients were coded as having been hospitalized for HF if there was at least one hospital stay with HF as the primary diagnosis. For patients with NT-proBNP at levels where it could make a difference according to the selection criteria depending on recent hospitalization, the electronic records were searched manually. For parameters with multiple values, the last available value was used for further analysis—in most from cases outpatient follow-up visits. Due to how the hospital's health care region stores lab results, ECGs, echocardiogram results, and currently prescribed medications, those parameters where accessible from other clinics, e.g., primary care providers.

### 2.3. Selection Process

We applied the major inclusion and exclusion criteria used in DAPA-HF and EMPEROR-reduced trials on the entire HF population to identify eligible patients. Some major inclusion criteria were the same in both studies: age 18 or older, left ventricle ejection fraction (LVEF) of 40% or less and New York Heart Association (NYHA) class of II-IV, and guideline-based individually tailored treatment with appropriate drugs and devices. Both studies required an elevated level of N-terminal B-type natriuretic peptide (NT-proBNP), but the cutoff level differed. DAPA-HF required NT-proBNP above 600 pg/ml, or at least 400 pg/ml, if the patient had been hospitalized for HF within the last 12 months. If the ECG showed atrial fibrillation or flutter at inclusion the threshold was at least 900 pg/ml even if they had a recent hospitalization, EMPEROR-reduced used a stratified cutoff value where patients with LVEF ≤ 30%, LVEF 31-40%, and hospitalization within 12 months were required to have NT-proBNP of at least 600 pg/ml. Patients with EF 31-35% were required to have NT-proBNP of at least 1000 pg/ml, and patients with LVEF of 36-40% were required to have at least 2500 pg/ml. Minimum levels of NT-proBNP were doubled if the patient's ECG showed atrial fibrillation at inclusion (to at least 1200, 2000, and 5000 pg/ml, respectively). DAPA-HF additionally reported major exclusion criteria in the form of recent treatment with, or unacceptable side effects associated with, an SGLT-2 inhibitor, type 1 DM (T1DM), symptoms of hypotension or a systolic blood pressure less than 95 mmHg, and an estimated glomerular filtration rate (eGFR) of less than 30 ml/minute/1.73 m^2^. Corresponding criteria according to EMPEROR-reduced differed in that the lower limit for systolic blood pressure was 100 mmHg, and the lower limit of eGFR was less than 20 ml/min/1,73 m^2^. Diabetes mellitus type 1 was not listed as an exclusion criterion for EMPEROR-reduced.

We used the revised Lund-Malmö method of estimating GFR [10, 11]. We chose this formula over CKD-EPI as the revised Lund-Malmö formula has shown better accuracy in this HF population [11]. NYHA-class was not routinely stated in medical records, and information regarding side effects from previous medication was not readily available for all patients; those criteria could not be applied. These modified criteria were applied to all patients who were alive on January 1^st^, 2020, using the latest available value for each parameter for the analysis.

### 2.4. Statistical Analysis

Variables with a normal distribution are reported as means with standard deviations. Nonnormally distributed variables are reported as medians with interquartile range. Categorical variables are described as frequencies with percentages. Group differences are compared with Student's *t*-test for continuous variables and *χ*^2^-test for categorical variables. We considered a *p* value <0.05 to be statistically significant, and we performed all analyses using SPSS version 27.

### 2.5. Ethics

This study complies with the Declaration of Helsinki. The Regional Ethical Review Board in Umeå, Sweden, has approved the study.

## 3. Results

### 3.1. Eligibility of SGLT2-Inhibitors in the Umeå Heart Failure Population

**Figure 1:**
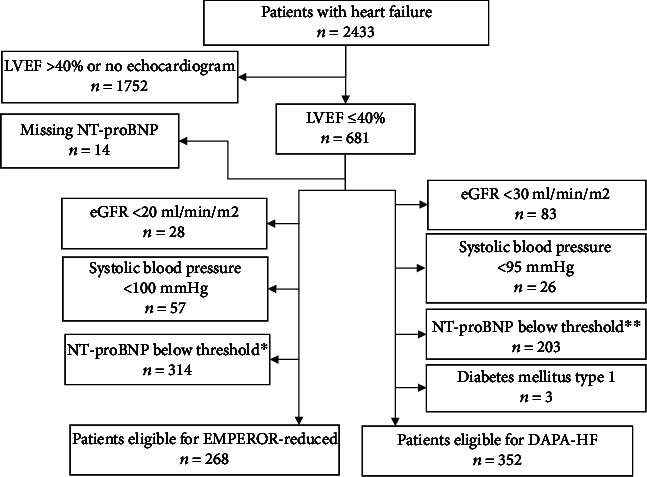
Selection of patients eligible for DAPA-HF and EMPEROR-reduced in the Umeå heart failure population when using applicable criteria from the DAPA-HF and EMPEROR-reduced trials. LVEF: left ventricle ejection fraction; eGFR: estimated glomerular filtration rate; NT-proBNP: N-terminal pro-B-type natriuretic peptide. ^∗^ < 600 pg/ml in patients with recent hospitalization or LVEF < = 30%, <1000 if LVEF 31-35%, and <2500 if LVEF 36-40%; doubled minimum values in patients with atrial fibrillation on latest ECG. ^∗∗^ < 600 pg/ml, or <400 pg/ml if hospitalization event for heart failure within last 12 months, or<900 pg/ml if last ECG showed atrial fibrillation or flutter.

Between January 2010 and December 2019, a total of 5162 patients aged 18 or older had been diagnosed with heart failure according to hospital registries. Of these, 2433 patients were alive January 1^st^, 2020 (suppl 1) but 106 were excluded because of missing echocardiogram data. A further 1645 patients were excluded due to a LVEF > 40%, leaving 681 (suppl 1) patients that met the major criteria of having a LVEF ≤ 40%, and were considered to have HFrEF. Eligibility status could not be determined for 14 (2%) of these patients due to missing NT-proBNP data (Figure 1). In 42 (10.6%) of the patients eligible for DAPA-HF or EMPEROR-reduced, the latest available NT-proBNP value was from an acute hospitalization event. The remaining 89.4% of values are from outpatient follow-up.

Out of the 681 patients with HFrEF, 352 (52%) met the eligibility criteria for DAPA-HF, corresponding to 14% of all patients with HF. The most common reason for ineligibility was a NT-proBNP below the threshold (*n* = 203), followed by renal impairment (*n* = 83) and hypotension (*n* = 26). Three patients were ineligible because of diabetes mellitus type 1. 15 patients met 2 exclusion criteria, 9 of which both had hypotension and too low NT-proBNP. No patient met 3 or more exclusion criteria.

For EMPEROR-reduced, 268 (39%) of the patients with HFrEF met the eligibility criteria, corresponding to 11% of all patients with HF. The most common reason for not meeting the eligibility criteria was NT-proBNP below the threshold (*n* = 314), followed by systolic blood pressure < 100 mmHg (*n* = 57) and eGFR < 20 ml/min/1.73m^2^ (*n* = 28). 32 patients met 2 exclusion criteria, of which 29 both had hypotension and too low NT-proBNP. No patient met all 3 exclusion criteria.

If eGFR was calculated with CKD-EPI instead of Malmö-Lund, 58 patients had eGFR < 30 ml/min/1.73m^2^, and 17 patients had eGFR < 20 ml/min/1.73m^2^.

**Figure 2:**
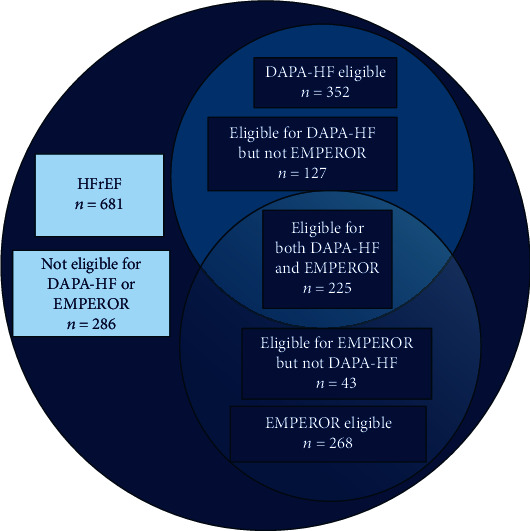
Distribution of patients according to eligibility status for DAPA-HF and EMPEROR-reduced.

Combining the eligibility criteria from both studies (Figure 2), 395 (58%) patients met eligible criteria for either study. Some patients met the DAPA-HF criteria but not the EMPEROR-reduced criteria (*n* = 127), while others met the EMPEROR-reduced criteria but not the DAPA-HF criteria (*n* = 43). Ignoring the NT-proBNP limits increases the proportion of eligible patients with HFrEF to 82.5% for dapagliflozin and 85.5% for empagliflozin.

### 3.2. Comparison between the Umeå HF Population and the DAPA-HF and EMPEROR-Reduced Populations

Characteristics for the patients in our population that were eligible for DAPA-HF and EMPEROR-reduced compared to the cohorts receiving dapagliflozin and empagliflozin in respective study are shown in Table 1. There were differences in most patient characteristics between our population and the trial populations, with the trial populations being numerically more like each other than either were to our real-world population. Our cohort was on average 12.8 and 13.1 years older than the DAPA-HF and EMPEROR-reduced populations, respectively (79.0 vs. 66.2 and 80.3 vs. 67.2 years) and had a higher proportion of women (32.6% vs. 23.8% and 34.7% vs. 23.5%). The prevalence of atrial fibrillation was higher in our population (56.0% vs. 36.1% and 53.0% vs. 35.6%), but the prevalence of DM was lower (21.0% vs. 41.8% and 26.1% vs. 49.8%). Renal function was worse in our population, with a lower mean eGFR (54.4 vs. 66.0 ml/min/1.73m^2^, and 49.5 vs. 61.8 ml/min/1.73m^2^) and more frequently had an eGFR < 60 ml/min/1.73m^2^ (67.9% vs. 40.6% and 73.9% vs. 48.0%). Hospitalization rates were similar in our patients compared to DAPA-HF patients (51.7% vs. 47.4%). EMPEROR-reduced reported hospitalizations within 1 year, but our data on timing of hospitalization event was incomplete and thus not comparable.

### 3.3. Comparison between Noneligible and Eligible Patients

Patient characteristics for patients deemed eligible for DAPA-HF compared to patients deemed ineligible are shown in Table 2. Ineligible patients are grouped according to which criteria caused them to be ineligible. We show the results for the two largest groups, i.e., the patients excluded due to renal impairment, and the group excluded due to NT-proBNP below the cutoff.

The group with low NT-proBNP was on average younger than the eligible group (70.9 vs. 79.0 years), had less comorbidities (rate of eGFR < 60 ml/min/1.73^2^ 36.9 vs. 67.9% and atrial fibrillation 32% vs. 56.3%), and was less often female (23.6% vs. 33.8%). They were less often treated with diuretics (47.3% vs. 66.2%) and more often had ICDs (25.6% vs. 13.4%).

The patients that were ineligible because of eGFR < 30 ml/min/1.73 m^2^ were older than the eligible patients (84 vs. 79 years), had a higher prevalence of T2DM (41.7% vs. 21%), and were less often treated with both ARNI (4.8% vs. 17.9% and MRA (41.7% vs. 60.6%), but more often had diuretics prescribed (90.5% vs. 66.2%).

## 4. Discussion

Our study of real-world patients with HF shows that about half of the patients with HFrEF would have been eligible for both major landmark clinical trials with SGLT2-inhibitors. DAPA-HF had slightly more generous inclusion/exclusion criteria than EMPEROR-reduced in regard to our population. Both trial populations differed significantly from our real-world population. The largest differences were that real-world patients were older, had worse renal function, higher rates of atrial fibrillation, and had a lower prevalence of DM compared to trial patients. Comparing the ineligible patients with eligible patients, it appears that the reason for exclusion reflects different parts of the patient and disease spectra, where both comparatively young and healthy patients and comparatively old and more morbid patients are excluded.

The eligibility rate seen in our population for these trials is comparable to what have been found for other HF treatments. Older studies on ACE-inhibitors, beta blockers, and MRAs have had eligibility rates among HFrEF-patients of 38%, 25%, and 55%, respectively [12]. Both DAPA-HF and EMPEROR-reduced have a higher eligibility rate than for sacubitril-valsartan according to PARADIGM-HF-criteria [13] in our population [14]. The eligibility rates for DAPA-HF and EMPEROR-reduced have been studied at other centers [15–17] in patients that are regularly followed up at a cardiology outpatient clinic. Our cohort includes a broader category of patients, as we have aimed to include all known HF patients in the community. A benefit to our approach is that the findings can be more generalizable to the entire community, which is of interest when analyzing cost-benefit for a new therapy. A drawback to our approach is less complete and stringent records on HF status. The eligibility rates we have found among HFrEF-patients are lower than have previously been reported, at 52% vs. 58-69% for DAPA-HF [15–17] and 39% vs. 50-53% for EMPEROR-reduced [16, 17]. Similarly to Maltês et al. and Monzo et al., our patients are significantly older with worse renal function than trial patients. Our population was less often treated with diuretics than either trial population or previously mentioned outpatient cohorts. This could mean that some of our eligible patients are asymptomatic and should have been classified as ineligible if NYHA-class was more often stated in records. In this case, the true eligibility rate would be lower.

The most impactful selection criterion was excluding patients with low levels of NT-proBNP. Neither the European Medicines Agency nor the United States Food and Drug Administration consider NT-proBNP levels in their approval text for either dapagliflozin [18, 19] or empagliflozin [20, 21]. Not taking NT-proBNP levels into account would mean treating a large group of patients that have not been represented in clinical trials. These patients also tend to be younger with less comorbidities according to our material.

Low eGFR was the second most impactful selection criterion for DAPA-HF and the third most impactful for EMPEROR-reduced, and overall, our population had significantly worse renal function than in either trial. Dapagliflozin has been studied in the setting of chronic kidney disease (CKD) with inclusion down to eGFR 25 ml/min/1.73^2^ [22], and both DAPA-HF and EMPEROR-reduced had renal outcomes as a secondary endpoint. Both SGLT2-inhibitors demonstrate a beneficial effect in the rate of decline for renal function and DAPA-CKD, a reduction in all-cause mortality. With the high overlap of HF and CKD seen in our population, SGLT2-inhibitors can serve a dual purpose in these patients. Patients with low renal function tend to be older and less likely to be treated with MRA or ARNI which emphasizes the need for new treatment alternatives for these patients.

Much of the differences between our population and the trial populations are attributable to the older age among our patients, since old age is a primary risk factor for both impaired renal function and atrial fibrillation. In a subanalysis of the older age group in the DAPA-HF trial, the patients had a prevalence of these conditions closer to what we have observed in our real-world study. This age group in the DAPA-HF had the same relative benefit of dapagliflozin as the younger patients. The absolute risk reduction was larger in the oldest patients, attributable to higher baseline risk [23]. This supports the conclusion that a real-world population like ours would benefit from SGLT2-inhibitor treatment despite being older and more comorbid. This is also important to consider when health economic analyses are performed as one can expect the absolute risk reduction in a real-world population to be greater than in the clinical trial.

Our population had a lower prevalence of T2DM than both DAPA-HF and EMPEROR-reduced. Previous observational data have shown a prevalence of diabetes in HF between 10% and 42% with the higher number derived from hospitalized patients and populations including HFpEF [24]. In DAPA-HF and EMPEROR-reduced, only patients with HFrEF were included, and they did not require hospitalization. One possible reason for the differences between our population and the study population may be that patients with known diabetes are easier to recruit to a HF study investigating the effects of a treatment already approved for diabetes.

### 4.1. Limitations

The retrospective study design based on medical record data is a limiting factor. In some cases, data was incomplete, and in other cases, the latest available ECG, echocardiogram, or blood test data was not up to date. With data from medical records, it was not possible to perfectly replicate the inclusion and exclusion criteria of DAPA-HF. Specifically, information of New York Heart Association (NYHA) classification was missing; however, both comparison trials included patients with NYHA classification II to IV, which represents most HF-patients—patients with NYHA class I have been reported to make up only 8-16% of HF patients [25, 26]. If the same proportions are true in our cohort, the true eligibility rate could be lower by a proportional amount. Additionally, since we used the most recent lab parameters, etc. to assess eligibility status, in 1/10 of eligible patients, the NT-proBNP value was from an acute hospitalization event which could lead to misclassification of some patients since acute hospitalization in associated with elevated NT-proBNP levels.

This is a single-center study, which limits the generalizability of our findings. However, our center is geographically the only hospital and cardiology clinic for the local population, and local guidelines posit that all HF be referred to the hospital for evaluation. This means that our material likely covers most known HF patients in the population at large.

## 5. Conclusions

In a real-world HF population, 52% of patients meet eligibility criteria for DAPA-HF and 39% for EMPEROR-reduced. The real-world patients are older than the trial patients, with a higher prevalence of renal impairment and a lower prevalence of diabetes mellitus. Trial entry criteria select for patients with a medium disease complexity, while excluding both the young and comparatively healthy, as well as the oldest and most ill patients.

## Figures and Tables

**Table 1:** Comparison of eligible patients for DAPA-HF and EMPEROR-reduced, compared to patients receiving trial drug in respective trial.

Characteristic	Umeå cohort eligible for DAPA-HF (*n* = 352)	DAPA-HF population (*n* = 2373)		Umeå cohort eligible for EMPEROR-reduced (*n* = 268)	EMPEROR-reduced population (*n* = 1863)	
			*p* value			*p* value
Age-yr	79.0 (±10.0)	66.2 (±11)	<.001	80.3 (±9.8)	67.2 (±10.8)	<.001
Female sex-no. (%)	119 (33.8)	564 (23.8)	<.001	93 (34.7)	437 (23.5)	
Body-mass index-kg/m^2^	26.4 (±4.9)^∗^	28.2 (±6.0)	<.001	26.3 (±4.8)^∗∗^	28.0 (±5.5)	<.001
Heart rate-beats/min	75.6 (±17.2)	71.5 (±11.8)	<.001	76.3 (±17.0)	71.0 (±11.7)	<.001
Systolic blood pressure-mmHg	124 (±18.2)	122 (±11.8)	.026	126 (±18.0)	123 (±15.9)	.002
Left ventricle ejection fraction-%	32.4 (±6.8)	31.2 (±6.7)	.002	30.1 (±6.7)	27.7 (±6.0)	<.001
Median NT-proBNP-ng/ml (IQR)	1943 (1138-3562)	1428 (857-2655)		2897 (1607-4872)	1887 (1077-3429)	
eGFR-ml/min/1.73 m^2^	54.4 (±15.0)	66 (±19.6)	<.001	49.5 (±17.8)	61.8 (±21.7)	<.001
Rate of eGFR <60 ml/min/1.73 m^2^-no./total no. (%)	239/352 (67.9%)	962/2372 (40.6)	<.001	198/268 (73.9)	893/1862 (48.0)	<.001
Hospitalization for heart failure-no. (%)	182 (51.7)	1124 (47.4)	.14	175 (65.3)	^∗∗∗^	
Atrial fibrillation-no. (%)	198 (56.3)	916 (38.6)	<.001	142 (53.0)	664 (35.6)	<.001
Diabetes mellitus-no. (%)	74 (21.0)	993 (41.8)	<.001	70 (26.1)	927 (49.8)	<.001
Hypertension-no. (%)	222 (63.1)	n/a		182 (67.9)	1349 (72.4)	.15
Ischemic etiology-no. (%)	163 (46.3)	1316 (55.5)	.002	130 (48.5)	983 (52.8)	.22
Heart failure treatment						
ACE-inhibitor or ARB-no. (%)	258 (73.3)	2007 (84.6)	<.001	195 (72.8)	1314 (70.5)	.50
ARNI-No. (%)	63 (17.9)	250 (10.5)	<.001	50 (18.7)	340 (18.3)	.94
MRA-no. (%)	213 (60.5)	1696 (71.5)	<.001	167 (62.3)	1306 (70.1)	.012
Beta blocker-no. (%)	322 (91.5)	2278 (96.0)	<.001	244 (91)	1765 (94.7)	.014
Diuretic-no. (%)	233 (66.2)	2216 (93.4)	<.001	204 (76.1)	n/a	
Digitalis-No (%)	52 (14.8)	445 (18.8)	.084	36 (13.4)	n/a	
ICD-no. (%)	47 (13.4)	622 (26.6)	<.001	38 (14.2)	578 (31.0)	<.001
CRT-no. (%)	53 (15.1)	190 (8.0)	<.001	46 (17.2)	220 (11.8)	.02

Number in parentheses is ±1 standard deviation; eGFR: estimated glomerular filtration rate; NT-proBNP: N-terminal pro-B-type natriuretic peptide; ACE: angiotensin converting enzyme; ARB: angiotensin receptor blocker; ARNI: angiotensin receptor blocker and neprilysin inhibitor; MRA: mineral corticoid antagonist; ICD: implantable cardioverter-defibrillator; CRT: cardiac resynchronization therapy. ^∗^BMI available for 333/352 patients. ^∗∗^BMI available for 255/268 patients. ^∗∗∗^DAPA-HF reports prior HF hospitalization, and EMPEROR-reduced reports hospitalizations in the last 12 months.

**Table 2:** Comparison of patients eligible for DAPA-HF compared to ineligible patient according to exclusion criteria.

Characteristic	Patients eligible for DAPA-HF (*n* = 352)	Patients not eligible due to NT-proBNP below threshold (*n* = 203)		Patient not eligible due to eGFR < 30 ml/min/m^2^ (*n* = 84)	
			*p* value		*p* value
Age-yr	79 (±10.0)	70.9 (±12.3)	<0.001	84.0 (±8.0)	<.001
Female sex-no. (%)	119 (33.8)	52 (23.6	0.02	33 (39.3)	0.41
Body mass index-kg/m^2^	26.4^∗^ (±4.9)	28.7^∗∗^ (±5.0)	<0.001	27.3^∗∗∗^ (±4.9)	0.13
Heart rate- beats/min	75.6 (±17.3)	71.3 (±13.9)	0.002	76.7 (±15.3)	0.2
Systolic blood pressure-mmHg	124 (±18.2)	125 (±16.8)	0.2	128 (±19.5)	0.6
Left ventricle ejection fraction-%	32.4 (±6.8)	34.8 (±5.6)	<0.001	32.9 (±6.5)	0.59
Median NT-proBNP-Pg/ml (IQR)	1943 (1138-3562)	286 (164-476)		3409.5 (1785-7073.75)	
eGFR-ml/min/1.73 m^2^	54.4 (±15.0)	65.5 (±17.8)	<0.001	21.5 (±5.9)	<0.001
Rate of eGFR <60 ml/min/1.73 m^2^-no./total no. (%)	239/352 (67.9)	75/203 (36.9)	<0.001	84/84 (100)	
Hospitalization for heart failure-no. (%)	182 (51.7)	102 (47.8)	0.42	39 (58.2)	0.2
Atrial fibrillation-no. (%)	198 (56.3)	65 (32.0)	<0.001	46 (54.8)	0.9
Diabetes mellitus-No. (%)	74 (21.0)	58 (28.6)	0.06	35 (41.7)	<.001
Hypertension-no. (%)	222 (63.1)	137 (67.4)	0.34	69 (82.1)	.001
Ischemic etiology-no. (%)	163 (46.3)	106 (48.2)	0.52	41 (48.8)	0.77
Heart failure treatment					
ACE-inhibitor or ARB-no. (%)	258 (73.3)	151 (74.4)	0.86	68 (81.0)	0.19
ARNI-no. (%)	63 (17.9)	45 (22.5)	0.27	4 (4.8)	0.005
Beta blocker-no. (%)	322 (91.5)	192 (94.6)	0.24	78 (92.9)	0.8
MRA-no. (%)	213 (60.6)	136 (67.0)	0.15	35 (41.7)	0.003
Loop diuretics-no. (%)	233 (66.2)	96 (47.3)	<.001	76 (90.5)	<.001
Digitalis-no. (%)	56 (15.9)	26 (12.8)	0.39	13 (15.5)	1
ICD-no. (%)	47 (13.4)	52 (25.6)	<.001	6 (7.1)	0.17
CRT-no. (%)	53 (15.1)	37 (18.2)	0.39	12 (14.3)	0.9

Number in parentheses is ±1 standard deviation; eGFR: estimated glomerular filtration rate; NT-proBNP: N-terminal pro-B-type natriuretic peptide; ACE: angiotensin converting enzyme; ARB: angiotensin receptor blocker; ARNI: angiotensin receptor blocker and neprilysin inhibitor; MRA: mineral corticoid antagonist; ICD: implantable cardioverter-defibrillator; CRT: cardiac resynchronization therapy. *p* values are in relation to the eligible patients. ^∗^BMI available for 333/352 patients. ^∗∗^BMI available for 197/203 patients. ^∗∗∗^BMI available for 82/84 patients.

## Data Availability

The spreadsheets and SPSS files containing the data used to support the findings of this study are available from the corresponding author upon request.

## Supplementary Materials

Supplementary Materials Characteristics for the cohort as a whole and the HFrEF population are shown in supplementary 1.
