# Supplementary material for: Eligibility of Dapagliflozin and Empagliflozin in a Real-World Heart Failure Population
Source: Cardiovasc Ther. 2021 Dec 26;2021:1894155. doi: 10.1155/2021/1894155 (PMC8720587; doi:10.1155/2021/1894155)
Supplement: Supplementary Materials — Characteristics for the cohort as a whole and the HFrEF population are shown in supplementary 1. [file 1894155.f1.docx]

| **Supplement 1: Baseline characteristics for all Umeå HF patients and HFrEF patients** | | |
| --- | --- | --- |
| **Characteristic** | **All Umeå HF patients**  (n=2433) | **All Umeå HFrEF patients**  (n=681) |
| **Age - yr** | 76.5 (±12.7) | 76.7 (±11.5) |
| **Female sex – no. (%)** | 1032 (42.4) | 208 (30.5) |
| **Body-mass index – kg/m^2^** | 27.9 (±5.7) | 27.2 (±5.0) |
| **Heart rate – beats/min** | 73.8 (±16.0) | 74.4 (±16.2) |
| **Systolic blood pressure – mmHg** | 128.5 (±20.0) | 123.6 (±19.1) |
| **Left ventricle ejection fraction - %** | 47.2 (±11.1) | 33.2 (±6.6) |
| **Median NT-proBNP – ng/ml (IQR)** | 883 (295-2147) | 1283 (484-3070) |
| **eGFR – ml/min/1.73 m^2^** | 55.4 (±19.9) | 54.0 (±20.6) |
| **Rate of eGFR <60 ml/min/1.73 m^2^ – no. (%)** | 1435 (59.0) | 418 (61.4) |
| **Hospitalization for heart failure – no. (%)** | 941 (38.7) | 355 (52.1) |
| **Atrial fibrillation – no. (%)** | 1182 (48.6) | 326 (47.9) |
| **Diabetes mellitus – no. (%)** | 651 (26.8) | 185 (27.2) |
| **Hypertension – no. (%)** | 1750 (71.9) | 456 (67.0) |
| **Ischemic etiology – no. (%)** | 955 (39.3) | 327 (48.0) |
| **Heart failure treatment** | |  |
| **ACE-inhibitor or ARB – no. (%)** | 1905 (78.3) | 505 (74.2) |
| **ARNI – no. (%)** | 148 (6.1) | 124 (18.2) |
| **MRA – no. (%)** | 1067 (43.9) | 410 (60.2) |
| **Beta blocker – no. (%)** | 2050 (84.3) | 631 (92.7) |
| **Diuretic – no. (%)** | 1343 (55.2) | 422 (62.0) |
| **Digitalis – no (%)** | 262 (10.8) | 101 (14.8) |
| **ICD – no. (%)** | 186 (7.6) | 116 (17.0) |
| **CRT – no. (%)** | 197 (8.1) | 111 (16.3) |
| *Number in parentheses are ± 1 standard deviation; eGFR, estimated glomerular filtration rate; NT-proBNP, N-terminal pro-B-type natriuretic peptide; ACE, angiotensin converting enzyme; ARB, angiotensin receptor blocker; ARNI, angiotensin receptor blocker and neprilysin inhibitor; MRA, mineral corticoid antagonist; ICD, implantable cardioverter-defibrillator; CRT, cardiac resynchronization therapy.* | | |
